# Intraventricular Silicone Oil: A Case Report

**DOI:** 10.1097/MD.0000000000002359

**Published:** 2016-01-08

**Authors:** Stéphane Mathis, Michèle Boissonnot, Jean-Pierre Tasu, Charles Simonet, Jonathan Ciron, Jean-Philippe Neau

**Affiliations:** From the Department of Neurology (SM, JC, J-PN); Department of Ophtalmology (MB); and Department of Radiology, CHU Poitiers, University of Poitiers, 2 rue de la Milétrie, Poitiers, France (J-PT, CS).

## Abstract

Intracranial silicone oil is a rare complication of intraocular endotamponade with silicone oil.

We describe a case of intraventricular silicone oil fortuitously observed 38 months after an intraocular tamponade for a complicated retinal detachment in an 82 year-old woman admitted in the Department of Neurology for a stroke. We confirm the migration of silicone oil along the optic nerve. We discuss this rare entity with a review of the few other cases reported in the medical literature.

Intraventricular migration of silicone oil after intraocular endotamponade is usually asymptomatic but have to be known of the neurologists and the radiologists because of its differential diagnosis that are intraventricular hemorrhage and tumor.

## INTRODUCTION

Rhegmatogenous retinal detachment occurs when there is a separation of the neurosensory retina from the retinal pigment epithelium with accumulation of subretinal fluid, in the presence of 1 or more retinal breaks: in the absence of treatment, it may cause severe visual loss.^1^ For ∼40 years, silicone oil is accepted as a safe and effective treatment used as an intraocular tamponade in the pars plana vitrectomy repair of retinal detachment; unlike gas tamponade, intraocular silicone oil injection allows prolonged tamponade because it is not absorbed. However, silicone oil can migrate into the anterior chamber of the eye and can accumulate in the peri-orbit (rarely in the subconjunctival space) after escaping the eye through sclerotomies.^2^ Intracranial migration of silicone oil has rarely been described. We report a fortuitous observation of intraventricular migration of silicone oil in a patient who was admitted for a stroke.

### Case Report

An 82-year-old Caucasian woman was initially admitted in our hospital for a sudden left hemiparesia. Her medical history consisted in atrial fibrillation (with anticoagulants) and cardiac pacing. She also suffered of a complicated retinal detachment on the left eye 38 months earlier (treated with intraocular silicone oil injection of 1300 centistokes): preoperative ocular was 22 mm Hg; because of this severe chronic retinal detachment (with retinovitreous retraction), precise visualization of the retina was difficult.

The clinical examination confirmed a motor mild weakness of her left hemibody. She did not complain of headache. Right eye vision and the cranial nerves were normal. General examination and skin examination were normal as well.

**FIGURE 1:**
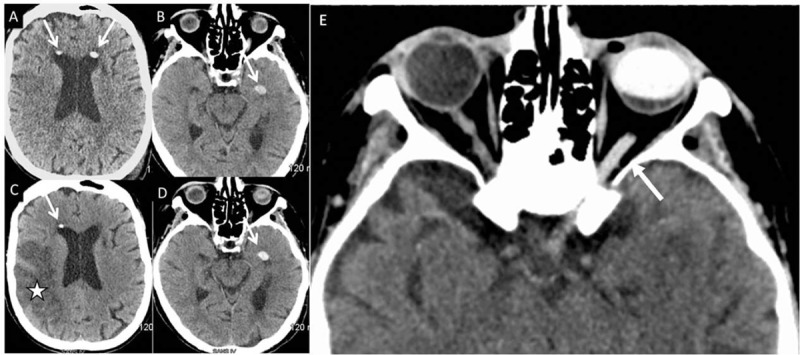
Day 0 (A, B) and day 2 (C, D) axial noncontrast-enhanced brain CT scans (same sections: A = C; B = D) showing moving intraventricular hyperdensities (arrows) and a cerebral infarction (star). E: hyperdensity of the left optic nerve (arrow) and the left ocular globe. CT = computed tomography.

Ancillary tests showed any abnormality. Because of the cardiac pacing, we were unable to perform a brain MRI. So, she underwent a brain CT-scan at admission: we observed 2 intraventricular spontaneous hyperdensities (Figure 1). Cervical extracranial arteries duplex sonography revealed a stenosis (> 70%) of the left internal carotid artery. Contrast-enhanced CT scan of the cervical arteries confirmed a stenosis (84%) of the left internal carotid artery. At day 2, we repeat the noncontrast-enhanced brain CT scan: we confirmed the infarct in the territory of the right middle cerebral artery, but also spontaneous migrations of the intraventricular hyperdensities (Figure 1). Silicone oil was visible in the left ocular globe (Figure 1*E*), with a density of 98 Hounsfield units (HU), but also along the left optic nerve (Figure 1*E*) where the density was 75 HU (in comparison of the right optic nerve where density was only 42 HU). For the spontaneous moving hyperdensities the density was quite the same that for the left ocular globe. These spontaneous hyperdensities were finally characteristic of the intraventricular migration of silicone oil. Ophthalmological examination confirmed ocular hypertension (35 mm Hg) without intraocular emulsified silicone oil. The patient died 3 months later of cardiac troubles.

## DISCUSSION

Although considered as a safe agent, the tolerance of silicone oil has been repeatedly questioned. It is well known that this foreign material may lead to serious intraocular complications (intraconjonctival oil inclusion cysts, band keratopathy, emulsification of oil with secondary glaucoma, cataract formation, and subretinal migration of oil), but also severe optic neuropathy caused by retrolaminar migration.^3^ In comparison with Schnabel's cavernous degeneration, a spongiform appearance of the proximal optic nerve due to focal loss of myelin and axons (with the preservation of septa), the process of infiltration of silicone within the optic nerve is called “pseudo-Schnabel's cavernous degeneration”: this phenomenon may be due to an increase of the intraocular pressure;^4^ it was also proposed that deep cupping of the optic disk may allow the silicone oil to enter the subarachnoid spaces (by breaking through the cerebral pia).^5^

By finding silicone bubbles in the optic nerve (and subarachnoidal spaces surrounding this nerve) after having analyzed an enucleated eye treated with silicone oil, it was confirmed that silicone may infiltrate the central nervous system.^6^ However, the frequency of the intracranial migration of intraocular silicone oil seems to be very low, as showed in a study where no case of intracranial silicone oil was observed in a series 19 consecutive patients several months (minimum delay of 2 months) after intraocular injection of silicone oil.^7^ To date, with ours, only 12 cases of intracranial migration of silicone oil were reported^5,8–17^ (Table 1). In 10 patients, the silicone oil was present in the ventricles, always in lateral ventricles and sometimes in the third (1 patient) and fourth ventricles (2 patients); only half of the patients (6) presented high density of the optic nerve (1 patient had optic nerve hyperdensity without intraventricular silicone oil); the time for the observation of silicone oil (mostly fortuitous) varied from 6 to 120 months. Sometimes, patients presented with a specific headache or dizziness; 1 patient presented with seizures. In all cases, there was no surgery.

**TABLE 1:**
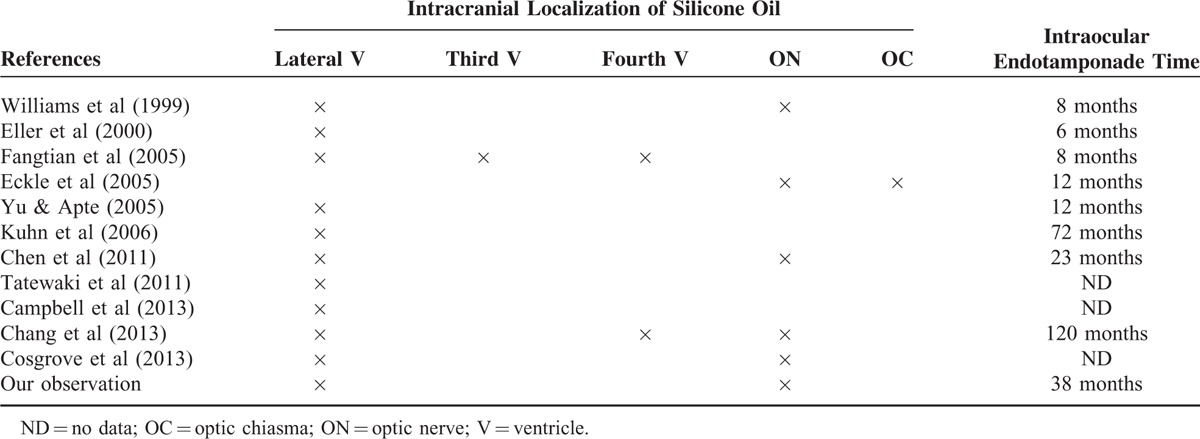
Cases of Intracranial Silicone Oil Migration After Endotamponade With Injection of Silicone Oil

In our patient, an intraocular silicone oil injection was performed 38 months earlier by using a particularly viscous fluid (1300 centistokes). On brain CT-scans the silicone oil was observed not only in the left ocular globe and the cerebral ventricles, but also along the left optic nerve, suggesting a migration of silicone oil in intracerebral ventricles along the optic nerve. Intraventricular silicone oil has a lower specific gravity as compared to cerebrospinal fluid (CSF), explaining its free-floating nature. Because of a high surface tension, its intraventricular configuration is usually spherical, conversely to hemorrhage that presents a fluid-fluid level.^14^ Silicone oil is transparent but radiodense, with a CT attenuation of 106 to 139 HU that is supposedly slightly higher than in hemorrhages (50–90 HU). However, and similarly to other clinical cases,^16^ even in the ocular globe this type of discrimination was not possible for us to achieve, given the fact that silicone oil density was inferior to 100 HU. One hypothesis to explain this relatively low density is a dilution of the silicone oil with CSF.^14^ Due to cardiac pacing, we were unable to perform brain MRI in our patient; that much said, brain imaging could confirm the previously mentioned migration diagnosis.^16^ Brain MRI may detect small droplets of silicone of 1 mm^3^ (or larger)^7^ that are hyperintense on T1-weighted images but with a variable signal intensity on T2-weighted images (iso-, hypo-, or hyperintense).^8^

Intraventricular migration of intraocular silicone oil has to be known of neurologists, ophthalmologists, and radiologists, even if it is of rare occurrence. It is a rare complication due to the migration of this material along the optic nerve, as shown in our case. Brain imaging may lead to the good diagnosis by demonstrating high attenuation on CT-scan (and hyperintensity on T1-weighted MRI) and a moving pattern when imaging is repeated: the recognition of this unusual combination of imaging characteristics may help to distinguish it from tumor or hemorrhage.

## References

[1] Saw SM Gazzard G Wagle AM An evidence-based analysis of surgical interventions for uncomplicated rhegmatogenous retinal detachment. *Acta Ophthalmol Scand* 2006; 84:606–612. 16965489 10.1111/j.1600-0420.2006.00715.x

[2] Nazemi PP Chong LP Varma R Migration of intraocular silicone oil into the subconjunctival space and orbit through an Ahmed glaucoma valve. *Am J Ophthalmol* 2001; 132:929–931. 11730665 10.1016/s0002-9394(01)01144-8

[3] Grzybowski A Pieczynski J Ascaso FJ Neuronal complications of intravitreal silicone oil: an updated review. *Acta Ophthalmol* 2014; 92:201–204. 23800347 10.1111/aos.12212

[4] Shields CL Eagle RC Jr Pseudo-Schnabel's cavernous degeneration of the optic nerve secondary to intraocular silicone oil. *Arch Ophthalmol* 1989; 107:714–717. 2719581 10.1001/archopht.1989.01070010732036

[5] Fangtian D Rongping D Lin Z Migration of intraocular silicone into the cerebral ventricles. *Am J Ophthalmol* 2005; 140:156–158. 16038670 10.1016/j.ajo.2005.01.006

[6] Papp A Toth J Kerenyi T Silicone oil in the subarachnoidal space—a possible route to the brain? *Pathol Res Pract* 2004; 200:247–252. 15200277 10.1016/j.prp.2004.01.001

[7] Kiilgaard JF Milea D Logager V Cerebral migration of intraocular silicone oil: an MRI study. *Acta Ophthalmol* 2011; 89:522–525. 20809907 10.1111/j.1755-3768.2009.01793.x

[8] Williams RL Beatty RL Kanal E MR imaging of intraventricular silicone: case report. *Radiology* 1999; 212:151–154. 10405734 10.1148/radiology.212.1.r99jl27151

[9] Eller AW Friberg TR Mah F Migration of silicone oil into the brain: a complication of intraocular silicone oil for retinal tamponade. *Am J Ophthalmol* 2000; 129:685–688. 10844074 10.1016/s0002-9394(00)00368-8

[10] Kuhn F Kover F Szabo I Intracranial migration of silicone oil from an eye with optic pit. *Graefes Arch Clin Exp Ophthalmol* 2006; 244:1360–1362. 16523301 10.1007/s00417-006-0267-9

[11] Eckle D Kampik A Hintschich C Visual field defect in association with chiasmal migration of intraocular silicone oil. *Br J Ophthalmol* 2005; 89:918–920. 15965179 10.1136/bjo.2004.062893 PMC1772707

[12] Yu JT Apte RS A case of intravitreal silicone oil migration to the central nervous system. *Retina* 2005; 25:791–793. 16141872 10.1097/00006982-200509000-00019

[13] Chen JX Nidecker AE Aygun N Intravitreal silicone oil migration into the subarachnoid space and ventricles: a case report and review of literature. *Eur J Radiol Extra* 2011; 78:e81–e88.

[14] Chang CC Chang HS Toh CH Intraventricular silicone oil. *J Neurosurg* 2013; 118:1127–1129. 23350782 10.3171/2013.1.JNS121570

[15] Tatewaki Y Kurihara N Sato A Silicone oil migrating from intraocular tamponade into the ventricles: case report with magnetic resonance image findings. *J Comput Assist Tomogr* 2011; 35:43–45. 21245688 10.1097/RCT.0b013e3181fc938d

[16] Campbell G Milbourne S Salman UA Ocular silicone oil in the lateral cerebral ventricle. *J Clin Neurosci* 2013; 20:1312–1313. 23688442 10.1016/j.jocn.2012.09.037

[17] Cosgrove J Djoukhadar I Warren D Migration of intraocular silicone oil into the brain. *Pract Neurol* 2013; 13:418–419. 24158510 10.1136/practneurol-2013-000715

